# Hints upon the Profession of Medicine

**Published:** 1851-09

**Authors:** Wm. Maxwell Wood

**Affiliations:** Surgeon U. S. Navy.


### BUFFALO MEDICAL JOURNAL

AND

### MONTHLY REVIEW.

VOL. 7. SEPTEMBER, 1851. NO. 4.

### ORIGINAL COMMUNICATIONS.

ART. I. — *Hints upon the Profession of Medicine.* By WM. MAXWELL WOOD, M. D., Surgeon U. S. Navy.

It is told of an ignorant but shrewd and impudent individual who became a wealthy and successful quack, that upon being called upon by a wondering friend, who had known him in his lowly estate, and asked how, with so few claims, he had risen to fame and fortune, he took his friend to the window which looked out upon a crowded London street, and asked how many wise or sensible men might be in the passing crowd? "Not more than one in a hundred," was the reply. "The remainder are mine."

The success of quackery, and the popular obstacles thrown in the way of true medical science being based upon those deficiencies of human judgment which are generally called human folly, and which, proverbially, form so large a portion of human nature, it is almost a hopeless task to correct the evil, and the attempt to do so may be only another manifestation of the general infirmity. Individuals who occupy positions of learning and influence, theologians and lawyers, and who in their general character have not the reputation of being either arrogant or conceited, constantly and confidently place their own views and opinions in opposition to those of the whole medical profession, and lend the influence of their names and position to the support of opinions which have not been to them a study, in opposition to the judgment of that profession which has made them a matter of most careful investigation. This fact proves that folly is not confined to the illiterate or to those who are marked specimens of imbecility, but that it pervades all classes, dilutes learning, and humiliates station. It shows also that the nature of the profession of medicine is not understood, and that the popular ideas in regard to it are formed from a most narrow and limited view of what constitutes the profession. These ideas are most probably derived from an acquaintance with but one class or division of the medical profession, that class which alone is brought into contact with the people. It is composed of those who pursue the profession as an art; its working men who laboriously and carefully drudge in the application of its means and details to their designed end. It may be that this acquaintanceship is limited to the medical working men of one locality, or at most of one country; and however able and skillful the individuals of this class may be, still they constitute but one, though an essential one of the many divisions which are embraced within the whole art and science of medicine. Again, the art of medicine is most probably understood as signifying only a mere routine acquaintance with the symptoms and names of diseases, and the application to those symptoms and diseases, of certain remedies which experience has taught to be available. Such limited views fall far short of the nature and scope of the profession of medicine. As well might the genius of Sir Humphrey Davy, the science which he adorned, and the philosophic inspiration which deduced the safety lamp, be sunk in the wire gauze of the little implement, or a view limited only to the miner who carries it. But were such views correct and sufficiently extensive, it would still seem that a decent modesty would teach those who feel the necessity of deferring to the judgment, in his vocation, of their tailor, shoemaker, or blacksmith, to pay a similar regard upon medical subjects, to those who have made them their study.

A very different, and much more expanded view is presented by the profession of medicine to one educated in it. He sees it in the various divisions, and vast arrangements spread out over the civilized world, and acting with all the power which can be derived from an aggregation of the highest orders of intellect, disciplined and strengthened to the utmost for its work. In every one of the various departments of his profession the medical student sees, not one only, but a collection of names designating individuals whose mental power demands the admiration of all who can appreciate their labors, —labors to which nothing short of the greatest intellectual strength is adequate. Follow then, the eye of this student as it sweeps over the cities of his own country, of England, France, Germany, Egypt, Prussia, and Turkey, and see with him the several divisions of the profession, studying man in health and disease from the microscopic elementary atom of each organ, up to his full development and arrangement in families, tribes, and nations.

Medical chemists, day and night, amid the machinery of their laboratories, are hunting nature in her hidden recesses, and exposing the principles and laws of combination. Medical microscopists are finding beauty of form and structure, where the naked eye sees not at all, or sees only a confused speck, and they are developing systems as wonderful in their minuteness, as that of astronomy in its magnitude. The anatomist, the physiologist, the pathologist, concentrating all their powers and observations upon the various subdivisions of these extensive sciences; the medical statistician, estimating the influence upon health and life of social and political conditions; of occupations; of population; of concentration in towns or diffusion in the country; the medical psychologist, studying the healthy and morbid manifestations of mind. Look, too, over distant parts of the globe, and see the medical corps of armies and navies adding to the common stock, their observations upon climates, the habits, diseases and remedies of different nations. Specially devoted to such observations and inquiries is the medical traveler, of whom the stationary practitioner may say, he

> " Seeks intelligence in every clime,
> And spreads the honey of his deep research
> At his return — a rich repast for me."

The professional inquirer finds in the large cities of Europe, Asia, and America, extensive hospitals and medical institutions: cities of disease in themselves; some of them devoted to a single variety of disease; and most of them presided over by men whose names have been for years before the profession associated with every constituent of professional greatness. These men, placed above the competition of general business, are occupied in gathering in discoveries, in trying supposed truths under every disadvantage, and with every precaution against fallacy, and then sending forth the proven results of their investigations. We must not, in this analysis of the components of the profession of medicine, lose sight of those professional martyrs, who, sacrificing all occupations of ease and profit, risk fortune, health, and life in the pursuit of scientific truth. Some inoculate themselves with disgusting and poisonous diseases; others pursue truth side by side with the pestilence until the fearful race terminates in death. In this general survey of his profession, the physician sees yet other important aids and appliances. Associations of medical talent issuing annual and learned treatises in their volumes of "Transactions," giving an abiding place to every established fac or subject for further investigation.— Medical conventions and national pharmacopœias, the latter treating of every remedy which has the least claim to respect, and the whole undergoing revision, addition, and improvement at stated periods. We have also before us medical missions and medical missionary societies, in the words of Professor Allison, of Edinburgh, establishing " the intimate connection that should ever subsist between the pursuit of scientific knowledge, and the reception of the blessed truths of Christianity."

The glance we have so far taken over the profession of medicine, rapid as it has been, is yet sufficient to show that it is not sought, in the records of Lord Bacon, " as a couch whereupon to rest a searching and restless spirit, or a terrace for a wandering and variable mind to walk up and down with a fair prospect, or a tower of state for a proud mind to raise itself upon, or a fort or commanding ground for strife and contention, or a shop for profit and sale, and not a rich store-house for the glory of the creator, and the relief of man's estate."

All these various fountains of knowledge are irrigating the whole profession by the channels of the medical press, and bearing to its humblest and most remote member whatever of fact, truth, and wisdom has been found worth the freighting. In the various modern languages we have, going forth, periodicals upon the profession in general, or upon branches of it. Most of these journals are in charge of men of eminent literary and professional ability, and any one number of either of the leading journals presents a specimen of intellectual ability of which not the profession only, but human nature may well be proud. Opening by chance one now lying on the table, we find thirty-two periodicals — English, French, German, American on its exchange list for the quarter. To present an idea of the scope of such journals, we find a single chance number to contain thirteen analytical and critical reviews of works in German, French, Italian, and English, the works being generally by the most distinguished medical authors of their respective countries. Then, besides medical reports, memoirs, and cases, we have, in the same number, fifteen bibliographical notices of works upon chemistry, diseases, anatomy, social problems, and natural theology: each notice being an interesting synôpsis of the work.

Could all see the profession of medicine even in the dim and feeble light by which we have endeavored to show its broad and comprehensive operations, the mind which could charge upon such a profession limited, selfish, and interested motives, opposed to truth, would only manifest its own incompetency to understand the nature and tendency of mental action, and nothing saves the many who utter such illiberal sentiments from contempt, but a general allowance for the blindness in which they are uttered — a blindness which must pertain to those not educated in the profession, and not living under its obligations. The professional man sees, with an enlarged vision, into regions closed to his unprofessional brother; in his discourse he proclaims the results of his vast system of moral, intellectual, and mechanical machinery, while the latter forms his positive opinions upon a few crude and disconnected facts, or supposed facts seen in the limited circle of his own untutored experience and observation. It is, as though one upon an eminence looks over a broad landscape, and speaks of its brilliant light, varied hues, and strong contrasts of color, while the man, blind from birth, obstinately contends that no such things exist, and that all is of one uniform darkness; or, it is, as though one by unaided vision sees only a blank speck in the intellectual atmosphere, while the telescopic aid of art and science shows it to be brilliant with glittering stones. Such considerations prevent us from impugning either the honesty, or the absolute intellectual ability of those who dogmatize boldly upon medical subjects, and though we must still wonder at their imagining themselves more familiar with medicine embracing a range of sciences, than with any other single science or language which they have not studied, the magnitude and boldness of their error become the measure of their ignorance and present their claim for forgiveness. For the benefit of all such, and for those who illiberally charge upon the medical profession a bigoted adherence to a limited system, or to selfish interests, inconsistent with its nature and mission, a few reasons will be presented why, upon the very constitution of human nature, such a limited action is morally impossible.

The love of truth is a principle implanted in the human mind, and which, in all ages, and in all sciences, has asserted its influence against every opposing circumstance. Under its direction men have surrendered every thing else dear to the human heart — suffered imprisonment, chains, ignominy, banishment, and death. None will deny these facts.

The science of medicine, in its very nature, must be under the influence of this principle, because it is the study of the Deity through his works. Here, then, we have a law of nature imposing upon the science of medicine a no less boundary than pure truth; and the next inquiry is whether the modes of medical investigation are those calculated to reach the truth.

We, in pursuit of the answer to this inquiry, see the human mind, such as it is, working in the science of medicine, not on a prescribed line, and up to a limited mark, but spreading out in every direction, according to its inclination and powers, every man according to his "proper gift of God, one after this manner, and another after that," and each class of laborers presenting its claim to the discovery of any truth, and finding reward and honor in the approbation of the mass of his co-laborers. Here, then, we have every guarantee that attainable truth shall be reached. We have the natural love of this virtue, we have the efficiency growing out of a chosen direction and concentration of power, we have scientific rivalry, vigilance and ambition, and, finally, we have the interests of every professional working man, stimulating him to make the acquaintance of these elaborated truths, and to apply them practically in the treatment of disease.

Whilst the profession of medicine is thus working in the main business of its vocation, it casts rich contributions upon subordinate and collateral sciences. To these sciences and their contributions the popular mind gives its admiration, and does not see that they are but chance jewels dug up on the way to the great mine. Look at Comparative Anatomy and Chemistry, Botany and Zoology, Geology and Mineralogy, and, in the words of a distinguished medical writer, "strip these sciences of what has been contributed to them by physicians, or by those who have had the discipline of a medical education, and a chasm is left which it would be difficult to fill."

A profession such as this is, rests upon principles far above the dogmas of any master-school, or Sir Oracle. It has every protection against fallacy which human reason can know, and is alike independent of the eccentricities of brilliant individual genius, and of the follies and vices of its unworthy members. Whilst there can be no influence sufficiently extensive to warp the common sense and common judgment of all, the intellect of any single individual may be subjected to honest or dishonest influences leading to fallacy or to wilful deception: hence the student of medicine learns, as the first step in the search after truth, to place no reliance upon individual authority, farther than such authority may be sustained by reason, and the testimony of established facts. Brilliant names, therefore, when they leave the proper ground of scientific truth to wander in the regions of fancy, are no authority for him, however imposing they may be upon the public. They are not suns, but meteors, and equally uninfluential is the clamoring testimony of the applauding crowds who may be dazzled by them. The medical student is too familiar with such things in his profession; and out of it he remembers that Hume and Gibbon were infidels, and that France dethroned christianity to set up the goddess of reason, and yet christianity resumed its place and power because it is true.

The misapplication or misunderstanding of a single word is often a fruitful source of a train of false ideas, false reasoning, and false conclusions. This is the case in regard to the word "system" as used in popular conversation upon medical science, and some considerations will now be presented upon this subject, which should be borne in mind by all desirous of forming a correct judgment upon the profession of medicine. The word "system," as applied to any medical dogma, theory, or scheme, on the one hand, and to the science of medicine on the other, naturally suggests the idea that the science of medicine is limited to some other and opposing dogma, or theory, to maintain which all its energies are directed. Hence, the advocates of any peculiar dogma are fond of using the terms — new system and old system — new school and old school — and by the acknowledgment of such distinctions, a whole train of error is founded. From what has already been said, the impropriety of such an application of terms, it is thought must be apparent. A science which seeks for truth cannot be limited by any system, but must pick up truth how and where it can; hence that of medicine in its very nature repudiates systems.

"In the present state of medical science, we feel well assured that the only true system is the absence of all systems. No premature attempt to generalize can have more than a temporary success. Be it ours to seek for light wherever it shall break in; to amass knowledge, even if we have to pick it from the mine; to draw wisdom from the errors and follies of our rivals, without disdaining to profit by their success;" and, then, "other systems will pass away, ours will be permanent; nourished, indeed, to some extent, by the very elements which come from their decay, as the eternal oak flourishes and grows green for ages from the decomposition of the transient vegetation, of which generations are springing up and perishing around it."

Before a correct and exact mode of investigation was established, all science, morality, and religion, were wrapped up in the dogmas of the schools and of the fathers, and men surrendered their minds to captivity and submission. Astronomy and chemistry, so exact now, wandered in the mazes of astrology and alchemy. Visionary theories agitated the profession of medicine; and as there was no law of truth and fact by which to try them, there was no limit to the wildness of unrestrained and imaginative intellects. Taught by such experience, all true science now repudiates systems and theories, or only recognizes them as based upon established facts, and as offering a reasonable supposition or direction for seeking the law of those facts — a conjecture as to the right road, formed upon the best existing knowledge, but which conjecture a little farther progress may show to be wrong.

That investigation which works within a system, has too limited and narrow a space to embrace the whole truth: a system can no more contain the whole science of medicine than a part can contain the whole. But in their subordinate relations all systems may be made tributary to science. For instance, a certain series of facts is observed, their phenomena, and the relations of these facts are seen and admitted by all — there is no dispute or difference in regard to them. Genius taking the facts endeavors to devise or discover a theory or law which shall embrace all of them. Different individuals suggest equally plausible theories, each has its partizans, investigation is carried on in various directions, to confirm the views of one set of theorists, and to confute those of others. In the progress of this investigation new facts are discovered which none of the theories will cover, and however satisfactory and beautiful they have, before this, appeared, they must now give way to those having a broader foundation in truth.

The entire profession of medicine may, then, be in accord as to certain facts, but may differ as to the general law influencing these facts. The facts alone are part of the profession. Such theories are very different from the wild schemes and visions which are engendered in some individual brain, and then the facts to sustain them imagined or asserted. Fever and inflammation have both afforded the material for many theories, and yet the facts constituting these diseases have been apparent to all. Difference of theory does not necessarily imply difference of treatment; indeed, the treatment of a disease upon which all are agreed, may be one of the facts upon which is founded different theories as to the nature of the disease. One set of theorists contend that the phenomena of inflammation depend upon an increased action of the blood-vessels of the part, and hence the heat, swelling, redness, and pain; other theorists say that these symptoms are the result of a diminished action, a want of tone in the vessels, in consequence of which the blood accumulates and stagnates in them. Both theorists, however much they may argue about about their opinions, agree upon their remedies, but one explains their action by saying that the remedies allay excitement, diminish tone, and the other contends that they impart strength and power to the debilitated vessels.

Many of the theories and systems in medicine have been splendid monuments of the power and wealth of the human mind; but as the rich materials of their creation lie scattered in ruin, they emblem the fallibility of the most exalted human intellect, and show that no genius can dare to leave the foundation of fact and truth, and yet hope to erect a firm and enduring structure. The science of medicine sits in judgment upon them all, and examines the claim of each in calm and philosophic impartiality, but refuses to any the privilege of fastening its link upon the chain of established law, until it presents that link in all the unyielding firmness and crystal transparency of truth.

The science of medicine, then, by its very nature, by the principles which govern the human mind, by every stimulus of interest and ambition, can limit itself to nothing short of attainable truth, and it cannot be limited by, or bound to any system. In the science of medicine there can be no "old school" or "new school," and the use of such terms creates a false impression, and misleads the popular judgment in regard to any scheme or pretension which aspires to independence of the profession of medicine. It has been endeavored to set forth the means, powers, combinations, and appliances necessary in so extensive an inquiry as that of professional truth, and they are seen to be such as impart efficiency to the human intellect and offer the best guards against fallacy. The decisions of such a scheme of mental and scientific operations ought surely to claim respect and confidence from the popular mind. History has sustained its decisions upon bygone medical delusions, no matter how strongly those delusions have been supported by popular enthusiasm, and it is certain that what the science of medicine now pronounces to be delusions will prove to be such. If any system or scheme sets itself above that professional investigation which is bound to seek for truth, and claims to be a new school on system, the claim and the pretension are alone proof that it is not true. If it contains any grain of truth, according to the laws which mind must obey that single grain must be found and added to the stores of general medical science. It is no argument to say that any "new school" system or sect has its colleges, hospitals, and journals, independent of general medicine. If such is the case it only proves that the disciples of such an arrangement have shut themselves within a narrow circle, and prohibited themselves from the broad search for truth, wherever it may be found; they have bound themselves to a one-man dogma, to a system, and not to a science. As well might theology be taught by schools of Swedenborgianism and Mormonism; and if they become schools of general science and theology, they cease of course to be those of a sect.

Persons who regard with favor some new scheme or pretence in the art of healing, strengthen their faith, and justify their opinions, by pointing to people in the respectable ranks of society who have given it their support. It may be that they name those distinguished in the literary world, or famed for their eloquence in the pulpit, and ask triumphantly, If it be not true, would such intelligent persons as these be found supporting this system? It is, perhaps, too common a mistake to imagine, because some particular merit, or accidental circumstance, elevates an individual to a superior station, that he necessarily has a correct judgment upon all matters. It may be that the very qualities which give him distinction, unfit him for close reasoning, or accuracy of judgment. If an excursive imagination, ingenious speculations, or that faculty of view and argument which can make the bad appear the better reason, are the causes of distinction, the chances are that the individual would be a bad interpreter of the truth, even were he acquainted with the particular science in which it is sought. But when an individual, unacquainted with any one of the series of sciences which constitute the profession of medicine, undertakes to give the support of his name and opinions to some system in opposition to general medical science, this act proves him to be wanting in common intelligence and correct judgment, whatever may be the general intelligence of the class or occupation he represents. His course is no less absurd than would be that of an individual who, without any knowledge of the sounds, letters, construction or meaning of a language, should dispute the interpretation of that language by those skilled in its knowledge.

The adherents of any special system, whether they may be its professors or its disciples and admirers, are incompetent to judge of truth independent of their system. Their minds are filled with that and nothing else. The student of general medical science sees, that while the stream of scientific truth has pursued its steady course from age to age, many such systems, schemes, and wild imaginings, have risen on its banks, and attracted the clamoring admiration of unthinking multitudes, whose fidelity only endures until a new pretence raises them from that which preceded it. The student of general medicine sees these peculiar notions in every age, making the same claims, presenting the same evidence, sustained by the same enthusiasm, and pointing among their followers to persons of the highest rank and respectability, and pretensions to intelligence.

If the retrospect runs back into the days of classic antiquity, pagan gods are seen to have been infallible physicians, with emperors for their patients, and whole nations testifying to their skill and success. In more modern times and countries, the altars of saints are seen covered with votive offerings in wax, silver or gold, representing arms, legs, hearts, heads, and whole figures of those whom the saint has cured of disease. The medical student can refer to one pill whose virtues were testified to by seventeen earls, eight viscounts, seventeen lords, fifteen bishops, six right honorables, seventeen coronets, five reverends, and many members of parliament, and yet the name of that pill is no longer heard.

When any individual representing a respectable station in society is asked to give his name to the support of quackery, or novel and peculiar systems, let him remember that while he may be flattered by his importance in the eyes of charlatans, quacks, and pretenders, science smiles in pity and contempt, to see him registering his name among the long list of those who have certified to their own ignorance, vanity and folly.

Continuing our review of some of the prominent delusions to which reference has been made, it is remembered that all France was once mad after the quack Montacino, when all the continent with a Prussian empress and princess, were subject to the delusions of the count and countess Cagliostro, who professed to restore, not only health, but youth and beauty, and received five thousand louis dors as a single fee. Mrs. Mopps' "Crazy Sally," as she called herself, ruled the popular mind of England, patrician and plebeian, and drove once a week to the Grecian Coffee House in a coach and six, with outriders. "We all remember," says the historian of this folly, "that the absurdity and impracticability of her own promises and enjoyments, were by no means equal to the expectations and credulity of those who ran after her; that is of all ranks and conditions of people, from the lowest laborer or mechanic, up to those of the most exalted station; several of whom not only did not hesitate to believe implicitly the most extravagant assertions, of an ignorant, illiberal, drunken female savage, but even solicited her company — at least seemed to enjoy her society." Add to these, the advent of mesmerism, the reign of Perkinism and "tractoration."

No delusion of the present, however strongly it may be supported, has more general, more respectable, or more intelligent unprofessional advocates, than had those past medical schemes which all unite now in calling folly and delusion.

(*Concluded in our next.*)

